# Mapping and modeling *Dirofilaria *infections in Europe

**DOI:** 10.1186/1756-3305-7-S1-O20

**Published:** 2014-04-01

**Authors:** L Rinaldi, V Musella, G Marzatico, M Mortarino, Genchi Claudio, G Cringoli

**Affiliations:** 1 Unit of Parasitology and Parasitic Diseases, Department of Veterinary Medicine and Animal Productions, University of Naples Federico II, Naples, Italy; 2 Department of Health Sciences, University Magna Graecia of Catanzaro, Italy; 3 Department of Veterinary Science and Public Health, University of Milan, Italy; 4 CIRPAR, Italy

## 

Climate change and increasing temperatures are a global phenomenon that can influence the dynamics of a number of hematophagous arthropods, vectors of pathogens with importance in human and veterinary medicine. In fact, climatic changes, together with an increase in the movement of dogs across Europe, have caused an increase in the geographical range of *Dirofilaria immitis *and *D. repens *infections. A Geographic Information System based on thermal regimen was constructed to identify areas potentially suitable for *Dirofilaria *transmission in Europe. These models are based on evidence that: i) there is a threshold of 14 °C below which *Dirofilaria *development will not proceed in mosquitoes; ii) there is a requirement of 130 growing degree-days for larvae to reach infectivity, and; iii) there is a maximum life expectancy of 30 days for a mosquito vector. The output of these models predicted that the summer temperatures (with peaks in July and August) are sufficient to facilitate extrinsic incubation of *Dirofilaria *even at high latitudes. Recently, an additional model was constructed to verify the influence of temperature in the course of three decades (1980-1989, 1990-1999 and 2000-2012) on the risk of infection by *Dirofilaria *in Italy. The results showed an expected increasing trend of temperatures, an increase of the *Dirofilaria *generation numbers into the mosquitoes and a significant extension of the infection risk from 5-6 months (1980-1989) to 6.5 months (1990-1999), up to more than 7 months (2000-20012). These findings show that geospatial tools are very useful for mapping, monitoring, forecasting and surveillance of both heartworm and subcutaneous dirofilariasis.

